# Disruption of *Visc-2*, a Brain-Expressed Conserved Long Noncoding RNA, Does Not Elicit an Overt Anatomical or Behavioral Phenotype

**DOI:** 10.1093/cercor/bhu196

**Published:** 2014-09-10

**Authors:** Peter L. Oliver, Rebecca A. Chodroff, Amrit Gosal, Benjamin Edwards, Amanda F.P. Cheung, Julio Gomez-Rodriguez, Gene Elliot, Lisa J. Garrett, Tom Lickiss, Francis Szele, Eric D. Green, Zoltán Molnár, Chris P. Ponting

**Affiliations:** 1 MRC Functional Genomics Unit; 2 Department of Physiology, Anatomy and Genetics, University of Oxford, Oxford OX1 3QX, UK; 3 Genome Technology Branch; 4 Embryonic Stem Cells and Transgenic Mouse Core, National Human Genome Research Institute, Bethesda, MD 20892, USA

**Keywords:** evolutionary conservation, knockout mouse, noncoding RNA, olfactory bulb, subventricular zone

## Abstract

Although long noncoding RNAs (lncRNAs) are proposed to play essential roles in mammalian neurodevelopment, we know little of their functions from their disruption in vivo. Combining evidence for evolutionary constraint and conserved expression data, we previously identified candidate lncRNAs that might play important and conserved roles in brain function. Here, we demonstrate that the sequence and neuronal transcription of lncRNAs transcribed from the previously uncharacterized *Visc* locus are conserved across diverse mammals. Consequently, one of these lncRNAs, *Visc-2*, was selected for targeted deletion in the mouse, and knockout animals were subjected to an extremely detailed anatomical and behavioral characterization. Despite a neurodevelopmental expression pattern of *Visc-2* that is highly localized to the cortex and sites of neurogenesis, anomalies in neither cytoarchitecture nor neuroproliferation were identified in knockout mice. In addition, no abnormal motor, sensory, anxiety, or cognitive behavioral phenotypes were observed. These results are important because they contribute to a growing body of evidence that lncRNA loci contribute on average far less to brain and biological functions than protein-coding loci. A high-throughput knockout program focussing on lncRNAs, similar to that currently underway for protein-coding genes, will be required to establish the distribution of their organismal functions.

## Introduction

The full range and complexity of RNA species that are transcribed in mammals are becoming increasingly apparent as a result of large-scale cDNA cloning and transcriptome sequencing projects (Okazaki et al. 2002; Carninci et al. 2005; Birney et al. 2007; Derrien et al. 2012). A substantial proportion of these transcripts are not translated: in addition to a myriad of small regulatory RNAs such as microRNAs (miRNAs), many thousands of long noncoding RNAs (lncRNAs) have been identified (Guttman et al. 2009; Derrien et al. 2012). These larger transcripts, defined as being over 200 nucleotides in length, are found not only within, or anti-sense to, protein-coding genes, but also in intergenic regions (Khalil et al. 2009). Despite the prediction that the number of distinct lncRNAs expressed in humans may surpass that of protein-coding genes (Derrien et al. 2012), very few of these have been studied experimentally in any detail and the molecular functions of only a handful have been described to date (Moran et al. 2012).

In the absence of abundant mechanistic findings, comparative evolutionary methods have been employed to show that evolutionary constraint within lncRNAs is generally low compared with protein-coding genes, even for closely related species (Ponjavic et al. 2007; Marques and Ponting 2009). For example, it has been estimated that 81% of human lncRNAs are confined to primates (Necsulea et al. 2014). However, some examples of conserved expression and functionality have begun to emerge (Chodroff et al. 2010; Ulitsky et al. 2011). In vitro studies, predominantly based on manipulating the expression levels of individual lncRNAs, have yielded some mechanistic insights and implicate these transcripts in diverse cellular processes (Wang and Chang 2011). Some lncRNA loci coincide with enhancer marks and their transcription is associated with modest increases in transcript abundance for neighboring protein-coding genes (Kim et al. 2010). Other lncRNAs act in trans via their interactions with intermediate nucleic acids or proteins (Moran et al. 2012; Vance et al. 2014). Potential functional significance has also been inferred from expression patterns of lncRNAs; for example, recent next-generation sequencing studies of mRNAs in the mammalian cortex have identified transcripts with a layer-specific distribution (Belgard et al. 2011).

To resolve the issue of functionality, it will be critical not just to consider lncRNA cellular roles and molecular mechanisms in the small set of chosen cell types that are assayed, but also to reveal their organismal functions by targeted disruption of their loci in model organisms. Only a relatively small number of mouse lncRNA knockout models have been reported thus far. For example, disruption of *Evf2* results in a subtle neuronal cellular phenotype (Bond et al. 2009) and loss of *Dlx1as*, the anti-sense lncRNA to transcription factor *Dlx1*, results in mild neurological defects (Kraus et al. 2013). Of 18 lncRNA knockout lines recently described, 5 were reported to have observable phenotypes including *Fendrr*, whose disruption was previously reported to result in embryonic lethality (Grote et al. 2013; Sauvageau et al. 2013). Reproducible cellular phenotypes have been observed in vitro by manipulation of *Neat1*, *Malat1*, and *HOTAIR* lncRNAs; however, no detectable physiological abnormalities were discernible in their corresponding constitutive knockout mouse models (Nakagawa et al. 2011, 2012; Schorderet and Duboule 2011; Eissmann et al. 2012; Zhang et al. 2012). In the case of *Malat1*, this was particularly unexpected given this transcript's exceptionally high level of expression reported in many tissues, including the brain, as well as evidence for a role of its lncRNA in pre-mRNA splicing recruitment and even cancer progression (Tripathi et al. 2010; Gutschner et al. 2013).

Without many further lncRNA knockout models, it would be premature to draw general conclusions from experimental data regarding the functional importance of lncRNA loci relative to protein-coding genes in mammalian systems. In addition, few studies have investigated the effects of targeted deletion of specific transcripts, while preserving other transcripts, in complex noncoding loci (Kraus et al. 2013). In this study, we investigated the phenotypic effect of disrupting a mouse lncRNA, *Visc-2*, from just such a complex locus. This transcript was chosen as it has been conserved in both sequence and brain expression across diverse mammals. We anticipated from its expression profile that effects of this disruption might be manifested in altered brain anatomy and/or behavior.

## Materials and Methods

### Multispecies Sequence Alignments

Regions orthologous to *Visc-1* and *Visc-2* (including the flanking 100 kb on each side of the indicated locus as shown below) were obtained from the following whole-genome sequence assemblies (available from the UCSC Genome Browser at http://genome.ucsc.edu): frog (*Xenopus tropicalis*; xenTro2), chicken (*Gallus gallus*; galGal3), songbird (*Taeniopygia guttata*; taeGut1), lizard (*Anolis carolinensis*; anoCar1), platypus (*Ornithorhyncus anatinus*; ornAna1), opossum (*Monodelphis domestica*; monDom4), mouse *(Mus musculus*; Mm9), rat (*Rattus norvegicus*; Rn4), guinea pig (*Cavia porcellus*; cavPor3), marmoset (*Callithrix jacchus*; calJac1), macaque (*Macaca mulatta*; rheMac2), orangutan (*Pongo abelli*; ponAbe2), human (*Homo sapiens*; Hg18), chimpanzee (*Pan troglodytes*; panTro2), horse (*Equus caballus*; equCab1), dog (*Canis familiaris*; canFam2), and cattle (*Bos taurus*; bosTau3). The liftOver program (Kent et al. 2003) was used to identify singly mapped (unambiguous) orthologous regions in the nonmouse species genomes. TBA (Threaded Blockset Aligner) was used to generate multisequence alignments (Blanchette et al. 2004) and visualized with Gmaj (Generalized Multiple Alignments with Java; Blanchette et al. 2004). Sequence identity plots were graphed with the program SinicView (Sequence-aligning INnovative and Interactive Comparison VIEWer; Shih et al. 2006). The corresponding genomic co-ordinates were selected: *A. carolinensis* scaffold_184:178408-1648757; *B. taurus* chr7:88511771-90384894; *C. jacchus* Contig1239:4-413851; *C. jacchus* Contig2424:1164-306723; *C. jacchus* Contig5:723588-1953659; *C. familiaris* chr3:22038316-23682511; *C. porcellus* scaffold_1:15464040-17198010; *E. caballus* chr14:78013389-79614242; *G. gallus* chrZ:58785269-59599138; *H. sapiens* chr5:86962138-88980753; *M. mulatta* chr6:83943471-85955231; *M. domestica* chr3:214376700-216926449; *M. musculus* chr13:82803274-85042441; *O. anatinus* chr1:5730158-7289157; *P. troglodytes* chr5:25947637-28003787; *Pongo abelii* chr5:88205383-90270057; *R. norvegicus* chr2:11050426-13768375; *T. guttata* chrZ:11967213-12578404; *X. tropicalis* scaffold_76:2296352-2888843.

### In Situ Hybridization

Target sequences were generated by PCR or reverse transcriptase (RT)–PCR, cloned into pCR4-TOPO (Invitrogen) and digoxigenin-labeled riboprobes were synthesized from linearized plasmid DNA. Tissue samples were snap frozen in OCT and 14 μm sections were cut using a cryostat (Leica) and mounted onto Superfrost Plus slides (VWR). Frozen sections of female human fetal frontal cortex (35 weeks gestation) were cut at 4 μm (Amsbio). Probe hybridization, washing, and signal detection using an alkaline phosphatase-conjugated anti-DIG antibody was carried out as previously described (Chodroff et al. 2010). Primer sequences for riboprobe cloning: *Visc-2* F 5′ GGATCACCATGACTGATCAG and *Visc-2* R 5′ ACCATTCTACCACTTGAGCC; *Visc-1* F 5′ GTTCACATCTCCTGCTTTCC and *Visc-1* R 5′ CCTGGAAACTTTCTATGTATT; *AK036048* F 5′ CTTCCTCGTAGAACACTGGAG and *AK036048* R 5′ CACCACACAGATTGCTAAGTG; *Visc-3* F 5′ CCTTATGCATTCTGCATTCATG and *Visc-3* R 5′ CTAGCTGAGCTACAGAATATC; Human *AK125167* F 5′ GTTTCGCGTGTTGTCAGCTG and *AK125167* R 5′ ATGGTACGAATATGGCTTCTTTC; Opossum *Visc-2* orthologue F 5′ YTATTGGATYACYATGTCTGATCAGCTG and 5′ CCAGTCTCYTCYAGGTMSTCACC.

### Quantitative RT–PCR and RACE PCR

qPCR analyses of *Visc-1*, *Mef2c*, *Tmem161b*, *Visc-3*, and *AK082072* were performed on a StepOne plus (ABI) using Power-SYBR master mix in a total volume of 20 μL. Measured fluorescence was normalized against GAPDH. Total RNA was extracted from whole brains using the RNeasy Mini kit (Qiagen) and 100 ng was reversed transcribed using Superscript II (Invitrogen) as per the manufacturer's instructions. Primers used were: *Visc-1* F 5′ GGACAACACTGCCTGGCGCAG and *Visc-1* R 5′ GGACAACACTGCCTGGCGCAGAC; *Visc-3* F 5′ CTGAGTCTTGCCTCGAGTATCTGTGC and *Visc-3* R 5′ GTGTCATTTGATCTGTCTGCGCCAGGC; *Mef2c* F 5′ CACGATGC CATCAGTGAATCAAAGG and *Mef2c* R 5′ GCCGAGGTGGAGCGCACTGGCAGTG; *Tmem161b* F 5′ GTTCTTGGCGTTATGGGTGTGATAGG and *Tmem161b* R 5′ TGCTTCCCTGCAAGGATTCTTAATTC; AK082072 F 5′ GTCAATAAAGGAATGTCCATCTG and AK082072 R 5′ CTAGAATTGGCATGGCATCACTC; *Gapdh* F 5′ TGTGTCCGTCGTGGATCTGA and *Gapdh* R 5′ CCTGCTTCACCACCTTCTTGA. All primers were assessed for efficiencies of the target and reference gene being approximately equal (absolute value of the log of the slope being <0.1). Four age- and sex-matched animals from each genotype were used and all reactions were performed in triplicate and analyzed using the comparative *C*_t_ method. Quantitative PCR analysis of *miR-9-2* was carried out using cDNA generated by the Taqman MicroRNA Reverse Transcription Kit (ABI). The Taqman Universal PCR Master Mix (ABI) was used with the snoRNA234 Taqman Small RNA Assays kit (ABI) as the reference, and data were analyzed using the comparative *C*_t_ method. 5′ and 3′ rapid amplification of cDNA ends (RACE) reactions were carried out using the RLM RACE system (Invitrogen) according to the manufacturer's instructions. PCR products from nested RT–PCR were gel-purified prior to sequencing.

### Generation of *Visc-2*^−/−^ Mice

pL452 and pL253 vectors were supplied by the Biological Resources Branch, NCI-Frederick Cancer Research, and Development Center (Liu et al. 2003). To insert components of pL452 into the bacterial artificial chromosome (BAC), the vector was first manipulated to include regions flanking the 5′ and 3′ ends of *Visc-2* by PCR. An approximately 800-bp fragment immediately upstream of *Visc-2* was cloned into pL452 upstream of the *loxP* site and *PGK* promoter. An approximately 800-bp fragment immediately downstream of *Visc-2* and within the first intron of *Visc-1* was also cloned directly downstream of *bGHpA* and a *loxP* site in pL452. The resulting pL452 components flanked by the 5′ and 3′ arms were removed by restriction digest and transformed into induced EL350 cells containing the RP23-451C5 BAC; incorporation of pL452 components into the BAC was confirmed by restriction enzyme digestion and sequencing (now termed BAC RP23-451C5-A). The targeting vector was also generated by recombineering using a modified pL253 vector containing homologous segments of RP23-451C5 flanking the region of interest. The required fragment was removed from pL253 by restriction digest and transformed into induced EL350 cells containing BAC RP23-451C5-A. The modified BAC was verified by restriction enzyme digestion and sequencing. Linearized DNA from this construct was transfected into ES cells by electroporation and screening for positive clones was carried out by Southern blotting of *ScaI*-digested genomic DNA. Eight-cell aggregations were carried out using embryos removed from pregnant BALB/c female mice and expanded C57BL/6J-derived, GFP-expressing mouse ES (LC3) cells; thus chimeric offspring could be identified by coat color or GFP expression. Chimeric mice were crossed with C57BL/6J wild-type mice, and transgene integration was confirmed by PCR using the primers WT_F (for the wild-type allele) 5′ AAAGAGCCCAGTCCCAATCT; HET_F (for the mutant allele) 5′ TCGACTAGAGCTTGCGGAAC and HET_WT_R (for wild-type and mutant) 5′ TGTCACCCCCAAATCATAGC.

### Animals

All mice used in this study were derived from *Visc-2*^+/−^ × *Visc-2*^+/−^ matings. Before dissection or behavioral testing, the mice were housed on a 12/12-h light/dark cycle (lights on at 0700 h and off at 1900 h). Mice were housed in groups of 4–5 with ad libitum food and water. All testing was performed during the light phase. The maintenance and testing of these animals were performed under the UK Home Office guidelines for the treatment of animals under scientific procedures and the local ethical review board at the University of Oxford.

### Neuroanatomical Screening

Adult (8 weeks of age [P56]) mice were perfused transcardially with 0.1 M PBS, followed by 4% paraformaldehyde (PFA). Brains were dissected out and post-fixed in 4% PFA at 4 °C overnight, then transferred to 30% sucrose for cryoprotection. Brains taken at P1 were dissected and immersion fixed in 4% PFA for 48 h prior to cryoprotection. Brains were cut at 30 μm using a freezing microtome (Leica) or at 20 μm using a cryostat (Bright). For Nissl staining (*N* = 3 *Visc-2*^+/+^ and *N* = 3 *Visc-2*^−/−^ at P56), every 12th section along the rostrocaudal axis was selected. For cortical layer marker studies (*N* = 3 *Visc-2*^+/+^ and *N* = 3 *Visc-2*^−/−^ at P1 and P56, respectively), equivalent regions of the somatosensory cortex were mounted on slides and blocked in 5% donkey or goat serum in PBS with 0.3% Triton X-100. Primary antibodies were incubated in the same blocking solution overnight at 4 °C: Nurr1 (1 : 50, R&D Systems), Foxp2 (1 : 1000, Abcam), Cux1 (1 : 50, Santa Cruz), Satb2 (1 : 100, Abcam), Tbr1 (1 : 400 Abcam), and Ctip2 (1 : 800 Abcam). Immunostraining for Nurr1, Tbr1, Satb2, and Ctip2 required additional retrieval in 10 mM sodium citrate pH 6.0 buffer (20 min) prior to blocking. The appropriate secondary antibodies (Alexa-488, Invitrogen) were applied for 2 h at room temperature.

For OB and rostral migratory stream (RMS) immunohistochemistry (*N* = 4 *Visc-2*^+/+^ and *N* = 5 *Visc-2*^−/−^), sections were counterstained with DAPI (5 μg/mL, Invitrogen) for 15 min (to allow confirmation of rostrocaudal levels), and blocked in 10% donkey or goat serum in PBS with 0.1% Triton X-100. Primary antibodies were incubated in the same blocking solution and incubated overnight at 4 °C: phosphohistone H3 (Ser10; pHH3; 1 : 500, Millipore), doublecortin (Dcx; 1 : 100, Millipore), glial fibrillary acidic protein (GFAP; 1 : 500, DAKO), calbindin D-28-K (CB; 1 : 2000, Swant), and calretinin (CR; 1 : 2000, Millipore). Secondary antibodies were applied for 90 min at room temperature: either donkey or goat anti-rabbit IgG Alexa-568 or -546 (Invitrogen), or for Dcx immunostaining, an additional biotin–streptavidin amplification (using biotinylated donkey anti-goat IgG [Abcam] and streptavidin Cy5 [USBiologicals]) was applied. To assess the subventricular zone (SVZ), 4 sections through levels within the range bregma 1.10–0.38 mm, interaural 4.90–4.18 mm (8 lateral ventricles in total per brain), were stained by immunohistochemistry. For quantification, the number of pHH3^+^ cells in the SVZ surrounding each lateral ventricle was counted. Note that pHH3 staining pattern depends on the phosphorylation state of the histone H3 protein, which changes during the cell cycle; only cells with uniformly intense nuclear staining (that reaches a maximum at metaphase) were included in the analyses. For assessment of the OB, 2 hemispheric sections from the mid-region of the rostrocaudal axis of the OB, and 2 from the more caudal OB, were used per brain. The number of CB^+^ cells in the glomerular layer was quantified by positioning four 200 μm × 200 μm boxes in this layer within each section, using Photoshop CS6. The same procedure was applied for CR^+^ cells; however, in addition 4 boxes were positioned in the granule cell layer of each section. For RMS analysis, 4 equivalent representative sections were taken from each brain and immunostained for pHH3 and Dcx.

For newborn neuron quantification in the OB, bromodeoxyuridine (BrdU) was injected intraperitoneally (i.p.) at 50 mg/kg once daily at P7 for 6 days (*N* = 4 *Visc-2*^+/+^ and *N* = 4 *Visc-2*^−/−^). After 14 days, the animals were perfused with 4% PFA, post-fixed in 4% PFA overnight, and cryprotected before cutting at 20 μm. Five equivalent coronal sections through the OB were first subjected to antigen retrieval (20 min in 10 mM sodium citrate pH 6) followed by water and PBS washes before blocking in 5% donkey serum with 0.3% Triton X-100. The sections were then incubated with sheep anti-BrdU (1 : 500, Abcam) made in the same blocking buffer overnight at 4 °C. After washing in PBS, donkey anti-sheep Alexa-488 was applied for 2 h. Co-staining for NeuN was carried out after additional blocking in 5% goat serum with 0.3% Triton X-100 using mouse anti-NeuN (1 : 500, Millipore) made in the same blocking buffer overnight at 4 °C. After washing in PBS, goat anti-mouse Alexa-594 was applied for 2 h before counterstaining with DAPI and mounting. For live counting of BrdU-positive (BrdU^+^) and BrdU/NeuN double-positive (BrdU^+^/NeuN^+^) cells, 5 fields of view at ×20 magnification were counted from both hemispheres from each of 5 equivalent sections from 4 mice of each genotype. All image quantification was performed blindly.

### Carbocyanine Dye Tracing

P7 mice were perfused with 4% PFA as above, the brains were dissected out and post-fixed in 4% PFA for 24 h, followed by long-term storage in 0.1 M PBS with 0.05% sodium azide. The brains were bisected along the coronal plane at the level of the posterior thalamus, so the cut surface contained ventrobasal complex, internal capsule, and the cerebral peduncle. Small crystals of the carbocyanine dye DiI (1,1′-didodecyl-3,3,3′,3′-tetra-methylindocarbocyanine perchlorate [(Invitrogen]) were placed under direct visual guidance, using a tungsten wire, into the cut surface of the thalamus (right hemisphere) and internal capsule (left hemisphere). We selected relatively larger crystals (up to ≈60 μm) for DiI placements into the cerebral peduncle and ventrobasal complex to achieve bulk labeling. Brains were incubated at 37 °C for 6 weeks according to previous protocols, to provide sufficient time for the diffusion of the dye within the cell membrane and accomplish both anterograde and retrograde labeling (Molnár et al. 2006). Following the incubation, the brains were embedded in 5% agarose (Bioline) and sectioned into 100 μm coronal sections on a vibrating microtome (VT1000S; Leica, Germany). They were counterstained with bisbenzimide (2.5 μg/100 mL, Hoechst 33258; Sigma) and mounted using 0.1 M phosphate buffer (PB) before being viewed on an upright epifluorescence microscope (Leica).

### Behavioral Testing

All behavioral testing was carried out on a cohort of 10-week-old male *Visc-2*^−/−^ and *Visc-2*^+/+^ (*N* = 12 for each genotype for each assay). Testing was carried out over a period of 4 weeks in the order described below.

#### Locomotor Activity

To measure novelty induced locomotor activity (LMA), experiments were conducted in the light phase between 1000 and 1400 h. Ambulations were measured in transparent plexiglass cages (20 × 35 cm), equipped with infrared photobeams (San Diego Instruments) over a total of 4 h.

#### Elevated Plus Maze (EPM)

The elevated plus maze (EPM) consisted of arms 30 cm long and 5 cm wide with the 2 opposing closed arms with 30 cm sides. Mice were placed in the EPM facing the open arm and allowed to freely explore the apparatus over 5-min trial using the Ethovision tracking software (Noldus). Entry into an arm of the EPM was defined using the base of the tail as the tracking position. The percentage spent exploring the open arms was calculated by dividing the time spent in the open arms by the combined time spent in open and closed arms.

#### Anxiogenic Open Field

Behavior was assessed in a white, anxiogenic open field (60 cm diameter, lighting at 3000 lux). An Ethovision software (Noldus) was used to split the arena into a central area of 10 cm radius and an outer area of 20 cm radius. Mice were placed into the apparatus at the edge and the distribution of time spent in the central and peripheral areas was taken as a measure of anxiety. The total distance traveled was also recorded.

#### Spontaneous Alternation in a T-maze

The apparatus consisted of a black T-shaped wooden maze made of arms, measuring 30 cm in length, 10 cm in width, and 29 cm in height. The goal arm entrances were provided with sliding guillotine doors. A central partition wall, extending 7 cm into the start arm, divided the choice point into 2 goal arms to prevent the mouse from receiving any sensory input from the nonvisited arm. A mouse was placed in the start arm of the T-maze and allowed to choose a goal arm. The mouse was then confined to the goal arm by sliding the guillotine door down. The arm entered (left or right) was recorded and the mouse was allowed to explore the goal arm for 30 s. The mouse was returned to the start arm, with the guillotine doors re-opened and the central partition removed, and allowed to explore again. The goal arm entered on the second run was recorded (left or right) and the mouse was then returned to its home cage. Each mouse was tested twice each day for 5 days (a total of 10 trials). The percentage of trials in which the mouse entered a different goal arm on the second run (i.e., alternated) was calculated.

#### Olfactory Preference Test

As a measure of the sense of smell olfactory response to both attractive and aversive scents was assessed as previously described (Witt et al. 2009).

#### Spatial Novelty Preference in the Y-Maze

Spatial novelty preference was assessed in an enclosed Perspex Y-maze with arms of 30 × 8 × 20 cm placed into a room containing a variety of extramaze cues. Mice were assigned 2 arms (the “start” and the “other” arm) to which they were exposed during the first phase (the exposure phase), for 5 min. This selection of arms was counterbalanced with respect to the genotype. Timing of the 5-min period began only once the mouse had left the start arm. The mouse was then removed from the maze and returned to its homecage for a 1-min interval between the exposure and test phases. During the test phase, mice were allowed free access to all 3 arms. Mice were placed at the end of the start arm and allowed to explore all 3 arms for 2 min beginning once they had left the start arm. An entry into an arm was defined by a mouse placing all 4 paws inside an arm. Similarly, a mouse was considered to have left an arm if all 4 paws were placed outside the arm. The times that mice spent in each arm were recorded manually and a novelty preference ratio was calculated for the time spent in arms [novel arm/(novel + other arm)].

#### Spatial Reference Memory: Morris Watermaze

The watermaze (diameter 2.0 m) was filled with water to a depth of 45 cm containing 500 mL of white poster paint to render the water opaque. In order to escape from the water the mice had to find a fixed location, hidden escape platform (diameter 21 cm) submerged approximately 1 cm below the water surface. The platform was located at the center of one of opposite quadrants (arbitrarily designated NW and SE). The number of mice trained to each platform position was counterbalanced with respect to the group. Animals received 4 trials per day for 9 days, with an intertrial interval of approximately 15 s. Mice were placed into the pool facing the side wall at 1 of 8 possible start locations (nominally N, S, E, W, NE, NW, SE, and SW, chosen randomly across trials), and allowed to swim until they found the platform, or for a maximum of 90 s. Any mouse that failed to find the platform within the allotted time was guided to the platform. The animal then remained on the platform for 30 s before commencing the next trial. The time taken to locate the platform and the distance traveled was quantified using the Anymaze (Stoelting) software. On the seventh (24 h after spatial training trial number 24) and tenth days of testing (24 h after spatial training trial 36), a probe trial was conducted to determine the extent to which the mice had learned about the spatial location of the platform. The platform was removed from the pool, and the mice were allowed to swim freely for 60 s. The percentage of time that animals spent in each quadrant of the maze was recorded.

### Genbank Submissions

BAC assemblies: spider monkey, DP001046; chicken, DP00104; echidna, DP001048; ferret, DP001049; gorilla, DP001050; guinea pig, DP001051; macaque, DP001052; marmoset, DP001053; opossum, DP001054; mouse lemur, DP001055; pig, DP001056; platypus, DP001057; zebrafinch, DP001058. RACE PCR of mouse *Visc-1*: KF032589 and KF032590.

## Results

**Figure 1.**
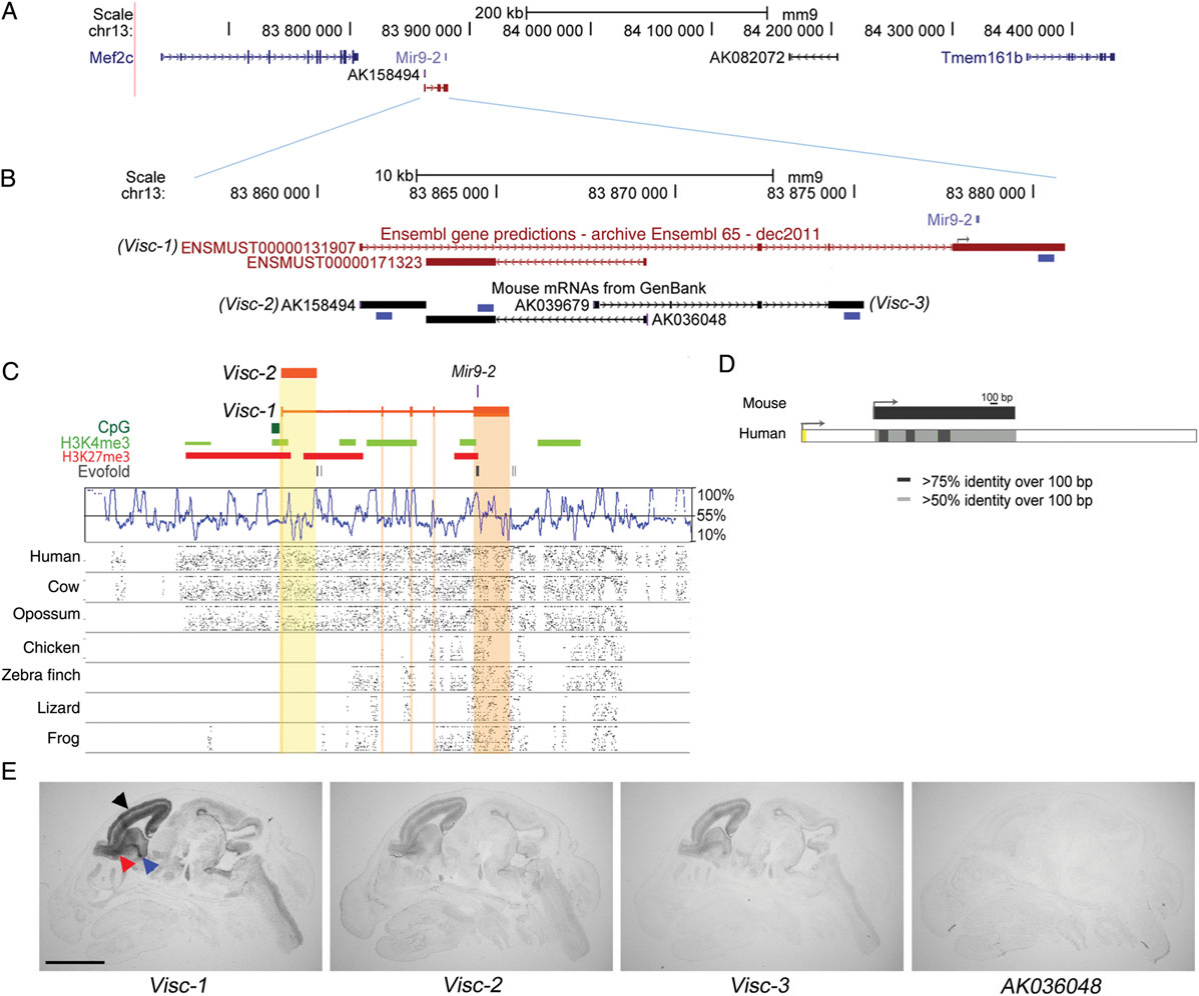
Genomic organization, conservation, and expression of the *Visc* locus. (*A*) The genomic region of mouse chromosome 13 (adapted from the UCSC Genome Browser), to scale, showing the relative position of *Visc-2* (*AK158494*). (*B*) Expanded view of the *Visc* locus, to scale. Blue boxes indicate the relative positions of the riboprobes used. Note that the major transcriptional start site in a human neuroblastoma cell line (SK-N-BE) lies 410 nucleotides from the miR-9-2 stem loop structure as shown (Laneve et al. 2010). (*C*) Expanded view of the *Visc* locus including H3K4me3 (green) and H3K27me3 (red) chromatin marks (Mikkelsen et al. 2007) and Evofold predictions of RNA secondary structures (dark grey). A SinicView conservation plot based on a 21-vertebrate sequence alignment is shown using mouse as the reference sequence, and Gmaj views of alignments between mouse and the indicated species’ sequences are also shown. (*D*) Conservation of the human *Visc-2* orthologue (*AK125167*). The TSS (arrows) and percentage sequence identity are indicated. (*E*) In situ hybridization against serial E16.5 wild-type mouse head sections shows the expression of lncRNAs in the *Visc* region. *Visc-1* is the most highly expressed, with hybridization to the telencephalon (black arrowhead), latero-caudal migratory path from the basal telencephalon to the striatum (blue arrowhead) and the RMS to the OB (red arrowhead). *Visc-2* and *Visc-3* (AK039679) expression share a virtually identical localization, although their levels of expression are lower. Expression of the lncRNA *AK036048*, on the opposite strand to *Visc-2*, is not detectable. Scale bar: 2 mm.

We previously identified the sequence of a brain-expressed lncRNA (accession number *AK158494*) as being highly conserved among mammals (Ponjavic et al. 2007). This single-exon lncRNA was identified originally from mouse visual cortex and was found among a set of cDNAs that were enriched for full-length sequences (Okazaki et al. 2002). Its locus overlaps with expressed sequence tags from cDNA libraries derived from both visual cortex and retina. The lncRNA is transcribed from an intergenic region of mouse chromosome 13 between *Mef2c* and *Tmem161b* (Fig. 1*A*). The *AK158494* locus partially overlaps with the first exon and intron of an Ensembl-predicted multi-exonic lncRNA ENSMUST00000131907 (Fig. 1*B*). We thus confirmed the expression and sequence limits of both these transcripts in the embryonic mouse brain using both RT–PCR and 5′/3′-RACE (*KF032589* and *KF032590*). Each of these lncRNAs was readily detectable, with the spliced variant showing 4 different canonical GT-AG introns, and both transcripts sharing a single putative promoter. In addition, both transcripts contain Evofold-predicted RNA secondary structures (Fig. 1*C*), but neither lncRNA contains an open reading frame that exceeds 100 amino acids. We shall refer to the locus as *Visc*, chosen to reflect the known expression of expressed sequence tags (ESTs) in the region in the *vis*ual *c*ortex, and to these 2 *Visc* lncRNAs as *Visc-1* (multi-exonic; ENSMUST00000131907) and *Visc-2* (single-exonic; *AK158494*).

DNA sequence of the *Visc* locus aligns with the genomes of evolutionarily distant vertebrates such as the frog *X. tropicalis* (Fig. 1*C*)*.* In addition, alignment of the mouse *Visc-1* multi-exonic sequence with multiple vertebrate genome sequences revealed that all donor and acceptor dinucleotide splice sites and a polyadenylation signal were conserved in evolution across mouse, human, and cattle, with only 2 substitutions in the metatherian opossum (*M. domestica*; Supplementary Fig. 1*A*). However, sequence conservation from mouse to opossum from the putative transcription start site (TSS) of mouse *Visc-1*/*2* was relatively modest (up to 66% sequence identity over 0.2 kb) and its sequence failed to align to nonmammalian vertebrate genomes. We therefore expected to identify only eutherian and metatherian *Visc-1* multi-exon orthologues, as well as *Visc-2* single-exon variants, possibly originating from distinct TSSs. A *Visc-2* orthologue was indeed identified in human (2.82 kb; *AK125167*), and as predicted, this transcript shares neither a TSS nor a termination site with its mouse counterpart, but it is 67% identical in sequence based on a local alignment (Fig. 1*D*). Sequence alignments also revealed human (3.4 kb; *LOC645323*), cattle (4 kb; *EE241071*), and pig (3.99 kb; *CK461258*) orthologues of *Visc-1* transcripts with a conserved exonic structure and sequence identities of 82% over 3.4 kb, 80% over 0.63 kb, and 86% over 0.73 kb, respectively (Supplementary Fig. 1*B*). Significantly, the tissue specificity of transcription appears to be conserved among species because these homologous cDNAs have been isolated from nervous system tissue (Supplementary Table 1). It is also noteworthy that conservation in the terminal 3′ exon of *Visc-1* is likely due, at least in part, to the presence of the micro (mi)RNA-9-2 (*miR-9-2*) precursor in its exonic sequence (Fig. 1*C*). *Visc-1* can thus be considered to be an mRNA-like noncoding RNA (Rodriguez et al. 2004), which is a miRNA precursor transcript, pre-miRNA9-2, in contrast to *Visc-2* which does not harbor miRNA sequences.

**Figure 2.**
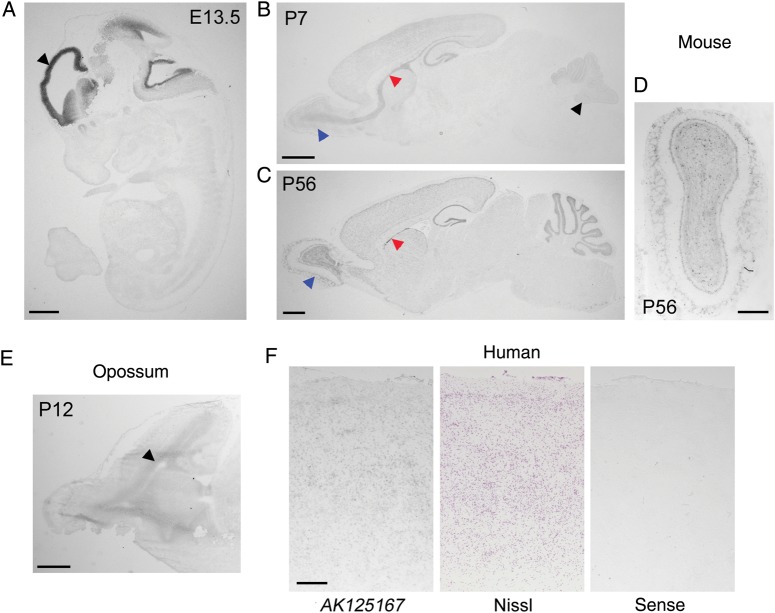
Developmental expression of *Visc-2* and orthologous lncRNAs in the brain. (*A*) In situ hybridization of E13.5 wild-type mouse embryos demonstrates localized expression of *Visc-2* in the telencephalon (black arrowhead). By P7, (*B*) expression is most prominent in the SVZ and OB (red and blue arrowheads, respectively), but also in the cerebellar granule cell layer (black arrowheads). Expression appears lower at P56 (*C*), but is still clearly localized to the proliferative regions of the SVZ and OB (red and blue arrowheads, respectively). A coronal view of the OB from a P56 wild-type brain is also shown (*D*). (*E*) Expression of the opossum orthologue of *Visc-2* was detected in the telencephalic ventricular zone (arrowhead) at P12. (*F*) Expression of the human *Visc-2* orthologue *AK125167* in the human fetal cortex compared with an adjacent Nissl-stained section and a section hybridized using a sense (negative control) riboprobe. Scale bars: 1 mm in (*A*–*C* and *E*); 0.5 mm in (*D* and *F*).

To characterize the tissue expression pattern of the *Visc* locus in more detail, in situ hybridization was carried out in the embryonic (E)16.5 mouse brain. Riboprobes were designed to investigate all of the annotated mouse lncRNAs in the region, including an additional transcript anti-sense (*AK036048*) and an additional splice variant, *Visc-3 (AK039679)*, which contains an alternative 3′ terminal exon (Fig. 1*B*). These data revealed that both *Visc-1* and *Visc-2* are preferentially expressed in the developing forebrain and are co-localized with the rostral and caudal interneuron migratory streams in addition to the cortical plate and the ventricular and subventricular zones of the telencephalon (Fig. 1*E*). To investigate the temporal distribution of this highly specific expression pattern, additional *Visc-2* in situ hybridization data were generated, showing that this lncRNA is expressed in the developing telencephalon at E13.5 but is not detectable outside of the brain (Fig. 2*A*). Furthermore, at early postnatal time points, expression is detected in all cortical layers, although it appears more pronounced in the neuroproliferative SVZ, RMS, and olfactory bulb (OB) as well as the granule cell layer of the cerebellum; this distribution continues into adulthood (Fig. 2*B*–*D*).

We have previously demonstrated that spatiotemporal expression of lncRNAs can be conserved in developing avian, marsupial, and eutherian brains (Chodroff et al. 2010). To determine whether there was similar evidence for *Visc-2*, expression of the orthologous opossum lncRNA was first confirmed by RT–PCR. Subsequently, in situ hybridization showed that expression of this lncRNA in the postnatal opossum brain occurs in neuroproliferative zones, including the telencephalic ventricular zone similar to that seen in the pre- and early postnatal mouse brain (Fig. 2*E*). We were, however, unable to identify a chicken orthologue for *Visc-2.* In addition, due to the apparent high level of sequence identity between mouse *Visc-2* and its human orthologue *AK125167* (Fig. 1*D*), we sought to confirm whether expression of the predicted human lncRNA was detectable by in situ hybridization. Fetal frontal lobe sections show the expression of *AK125167* in all cortical layers at an estimated developmental time point that can be considered equivalent to the early postnatal stage in the mouse cerebral cortex (Fig. 2*F*; Clancy et al. 2001). Taken together, these data provide evidence for evolutionary conservation in sequence and in the regulation of transcription of *Visc-2* across distantly related mammals, suggesting that this lncRNA plays an important and conserved functional role in the developing mammalian nervous system.

**Figure 3.**
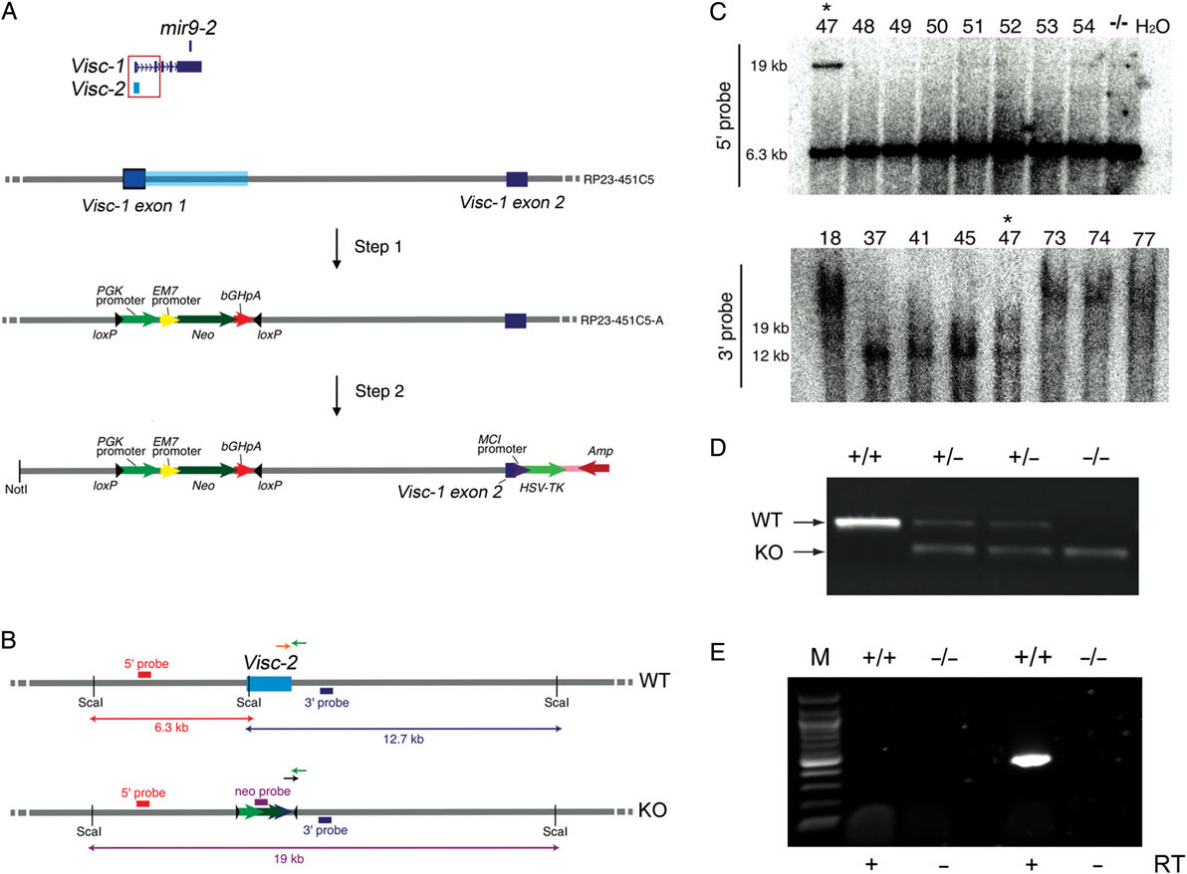
Generation of a *Visc-2* knockout mouse. (*A*) Overview of the generation of a *Visc-*targeting transgenic mouse constructs using BAC recombineering. The BAC RP23-451C5 encompasses both *Visc-1* exons 1 and 2 (dark blue) and *Visc-2* (light blue). In step 1, this region was replaced by components of the pL452 vector (including the *PGK* eukaryotic-derived and *EM7* prokaryotic-derived promoters that drive expression of the neomycin drug-resistance gene [*Neo*]) followed by the bovine growth hormone polyadenylation tail (*bGHpA*) to produce the modified BAC RP23-451C5-A. In step 2, the region of interest was subcloned into vector pL253 to create the *Visc-2* knockout targeting construct. This vector includes a polyoma enhancer promoter (MCI) driving expression of herpes simplex virus thymidine kinase (*HSV-TK*) with ampicillin (*Amp*) selection. (*B*) Mouse genomic region encompassing targeted region in wild-type and *Visc-2* knockout illustrating the probes for Southern blotting screening are indicated by red and blue boxes, respectively. Primers for PCR-based genotyping of the 2 alleles are shown as green and orange (*Visc-2*^+/+^ [WT, wild-type]); and green and black arrows (*Visc-2*^−/−^ [KO, knockout]). (*C*) 5′ and 3′ Southern blot screens showing positive homologous recombinant (indicated by asterisk, clone number 47), using *ScaI* restriction enzyme digestion of genomic DNA from targeted ES cells. (*D*) Genotyping of DNA extracted from *Visc-2*^+/+^, *Visc-2*^+/−^, and *Visc-2*^−/−^ mice. (*E*) RT–PCR of *Visc-2* from E16.5 whole-brain cDNA confirming the absence of the transcript in *Visc-2*^−/−^ mice. Control reactions in the absence of reverse transcriptase (-RT) during cDNA preparation are also shown. M, 100 bp marker.

To gain greater insights into the organismal function of *Visc-2* in vivo*,* a constitutive knockout mouse was generated. As the *Visc-2* transcript shares a predicted TSS with *Visc-1* (see above), our strategy to attenuate expression was to selectively delete the entire *Visc-2* locus (including the *Visc-1*/*Visc-2* TSS), with the expectation that transcription of both lncRNAs would be abrogated. A BAC clone spanning the first 2 exons of *Visc-1* was modified by recombineering and subsequently subcloned to create a targeting construct (Fig. 3*A*,*B*). This construct was electroporated into embryonic stem (ES) cells and homologous recombination was confirmed by Southern blotting (Fig. 3*C*). Aggregation of ES cells heterozygous for the targeting construct into 8-cell embryos produced one viable and fertile male mouse with an estimated high percentage of chimerism. Subsequent breeding from this chimera generated the expected Mendelian inheritance ratios of wild-type (*Visc-2*^+/+^), heterozygous (*Visc-2*^+/−^), and knockout (*Visc-2*^−/−^) offspring of both sexes (Fig. 3*D*; Supplementary Table 2). The loss of the *Visc-2* transcript was also confirmed by RT–PCR from the E16.5 brain (Fig. 3*E*). Initial examination of *Visc-2*^−/−^ mice revealed that they are fertile and appear to develop normally, with neither male nor female knockout animals showing significant differences in size, weight, or lifespan relative to *Visc-2*^+/−^ or *Visc-2*^+/+^ controls from birth to adulthood up to 18 months of age (data not shown).

**Figure 4.**
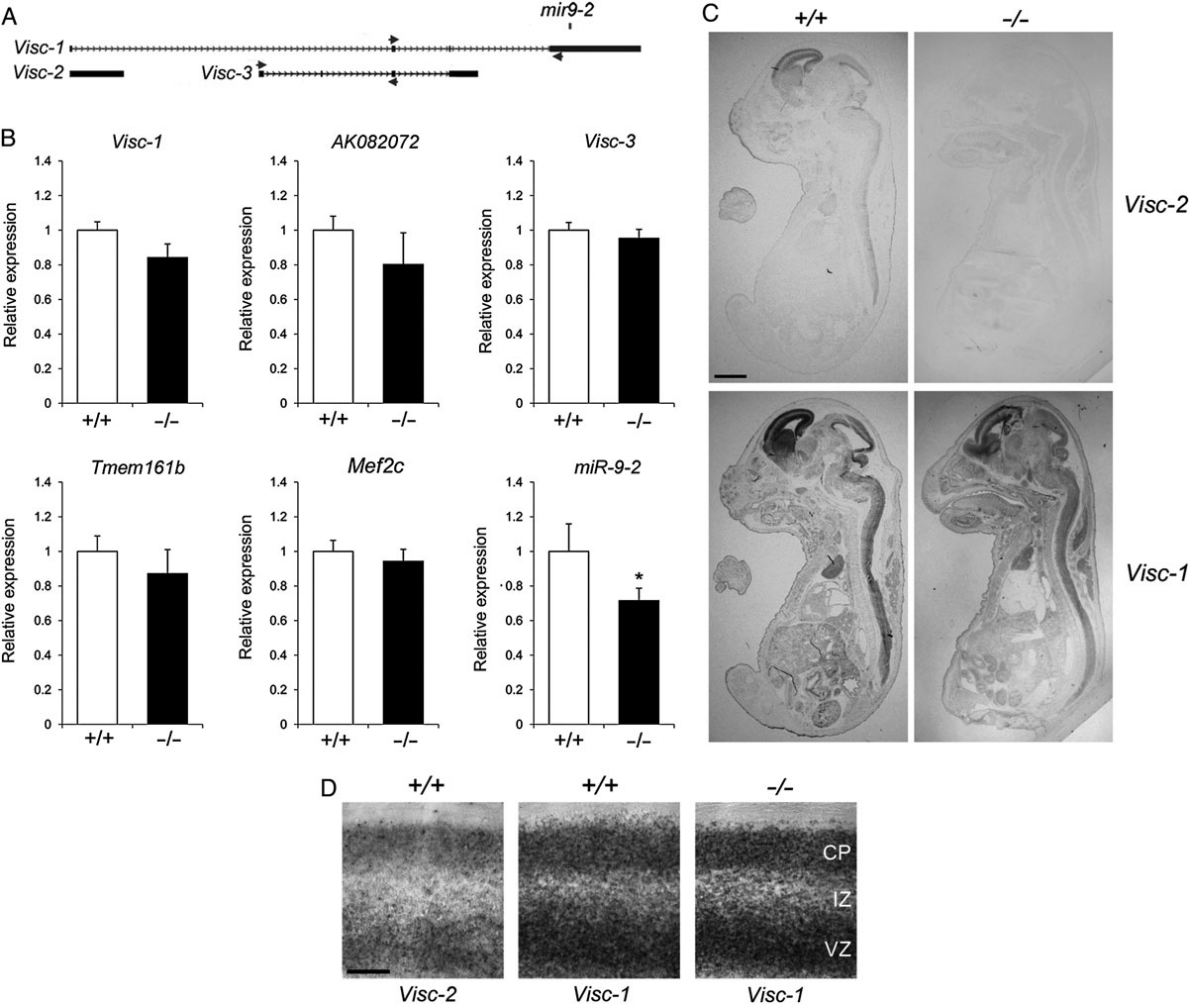
Expression analysis of protein-coding and noncoding transcripts expressed in close proximity to the *Visc-2* locus. (*A*) Detail of the *Visc* region, to scale, showing a relative position of the primers used for qRT–PCR. (*B*) Quantitative RT–PCR from E16.5 *Visc-2*^+/+^ and *Visc-2*^−/−^ whole brain. There was no significant difference between genotypes in the expression of protein-coding genes (*Tmem161b* [*P* = 0.14] and *Mef2c* [*P* = 0.26]), or the noncoding transcripts *AK082072* (*P* = 0.21), *Visc-1* (*P* = 0.17), or *Visc-3* (*P* = 0.34); however, a significant reduction in the expression of *miR-9-2* was observed (*P = 0.032; ANOVA). All data are shown ±SEM. (*C* and *D*) In situ hybridization using riboprobes specific for *Visc-1* and *Visc-2* at E16.5 showing loss of *Visc-2* expression in *Visc-2*^−/−^ whole embryos (*C*) and the developing cortex (*D*), but no disruption in the expression level or distribution of *Visc-1* in knockout mice. Scale bars: 2 mm in (*C*) and 0.25 mm in (*D*).

To assess whether the loss of *Visc-2* influenced the expression of genomically neighboring loci, qRT–PCR analysis of the E16.5 brain was performed to examine genes present in a large genomic region flanking the deleted locus (Figs 1*A* and 4*A*). These data show there to be no significant difference in the expression levels of flanking protein-coding genes *Tmem161b* or *Mef2c*, or a conserved lncRNA present over 200 kb downstream of *Visc-2*, *AK082072* (Fig. 4*B*). Unexpectedly, there was no influence of the deletion of *Visc-2* on the expression of *Visc-1* as shown by qRT–PCR (Fig. 4*B*) and in situ hybridization (Fig. 4*C*,*D*), indicating that alternative *Visc-1* RNAs are transcribed from additional promoters that are not shared with the deleted *Visc-2* locus. Nevertheless, expression level of the *miR-9-2* mRNA was significantly reduced by approximately 30% in knockout brain tissue at this time point (*P* = 0.032; Fig. 4*B*). Complete abrogation of expression of mRNA-9-2 in *Visc-2*^−/−^ mice was not expected because expression of a major transcript in human neuroblastoma cells requires a promoter only just upstream of the pre-miRNA 5′ end (Laneve et al. 2010).

An initial overview of the gross morphology of *Visc-2*^−/−^ brains by Nissl staining uncovered no abnormalities in structure or cytoarchitecture, compared with controls (Supplementary Fig. 2). Although no overt anatomical changes were observed, this warranted a more detailed regional study. Initially, considering the high levels of *Visc-2* in the developing cortex, markers with known layer-specific patterns of expression in this region were analyzed at early postnatal (P1) and adult (P56) in *Visc-2*^−/−^ and *Visc-2*^+/+^ mice (Supplementary Fig. 3). These data show normal distribution of markers in the subplate (Nurr1), through layers 4–6 (Ctip2, Foxp2, and Tbr1) to the upper layers 2–3 (Satb2 and Cux1) at both time points, suggesting that no overt patterning or migration defects occur in the cortex of *Visc-2*^−/−^ mice.

**Figure 5.**
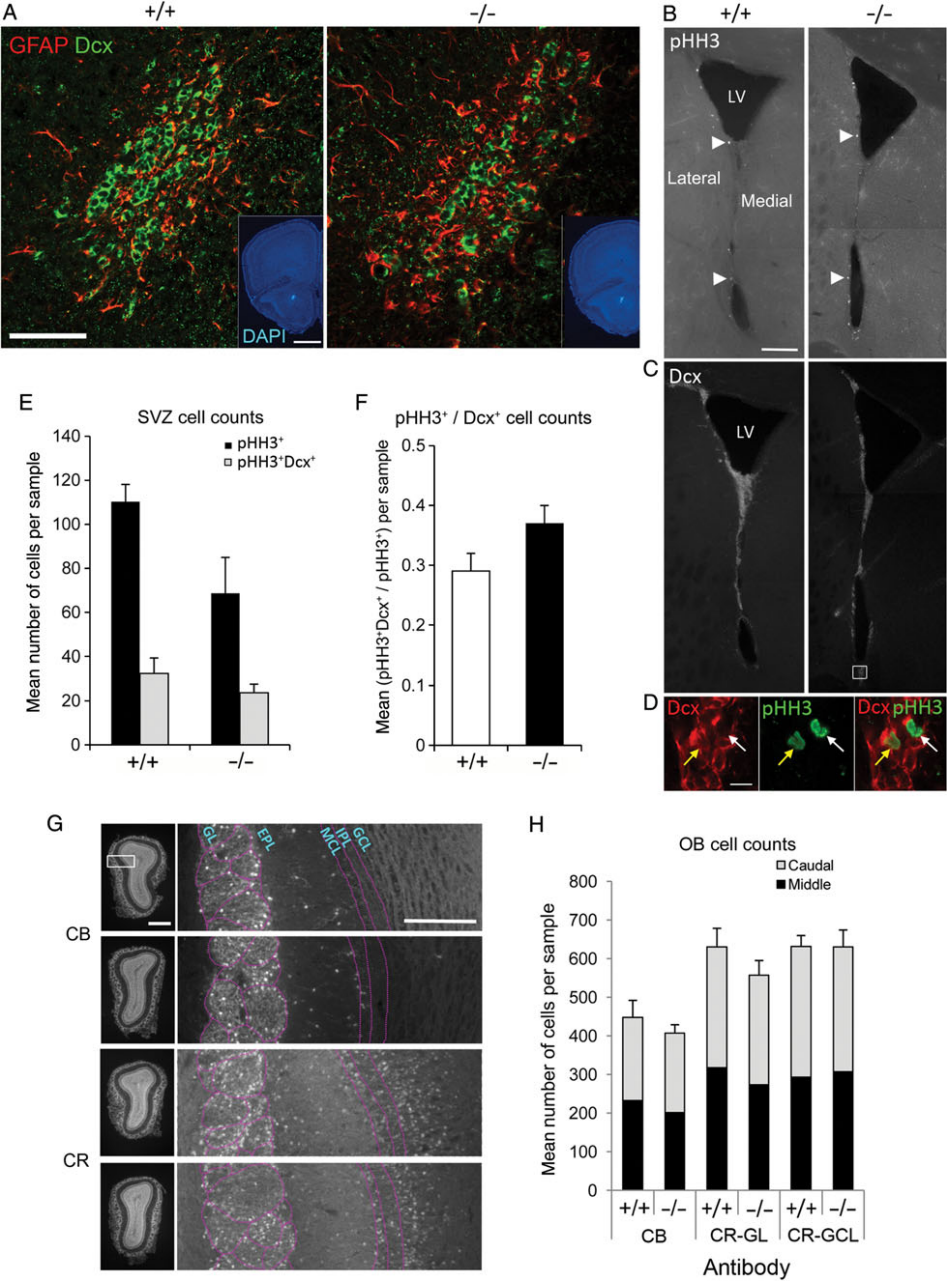
Neuroanatomical screening of the adult SVZ, RMS, and OB in *Visc-2*^−/−^ mice. (*A*) Representative confocal micrographs of the RMS illustrate the lack of colocalization between GFAP (red) and Dcx (green) cells indicative of normal structural integrity in both *Visc-2*^+/+^ (+/+) and *Visc-2*^−/−^ (−/−) animals. Scale bar: 50 μm. (Insets) DAPI-counterstained images indicating the position of the RMS, as shown in (*A*), respectively. Scale bar: 1 mm. (*B*) Montaged fluorescence micrographs showing the distribution of dividing cells within the SVZ in *Visc-2*^+/+^ and *Visc-2*^−/−^ mice. White arrowheads indicate example pHH3^+^ cells, which were largely located within the lateral wall of the lateral ventricle. Scale bar: 200 μm. (*C*) Montaged fluorescence micrographs showing the migratory neuroblast population within the SVZ, as shown by Dcx staining along the (mainly lateral) ventricular surface. LV, lateral ventricle. (*D*) Coexpression of pHH3 and Dcx labeling of the region indicated by the box in (*C*); cytoplasmic Dcx (red) staining of the neuroblasts, nuclear (green) staining of the proliferating cells. The merged image shows a coexpressing (yellow arrow) and a noncoexpressing (white arrow) cell. Scale bar: 10 μm. (*E*) Quantification of SVZ populations showed no significant difference in pHH3^+^ (*P* = 0.073) and pHH3^+^Dcx^+^ (*P* = 0.27) cells between genotypes or (*F*) proportion of pHH3^+^ cells that were co-labeled with Dcx (*P* = 0.14). Data are shown ±SEM. (*G*) Representative DAPI-counterstained fluorescence micrographs of sections taken from the mid-region of the OB from *Visc-2*^+/+^ and *Visc-2*^−/−^ mice; boxes show the position of the higher magnification images. The CB^+^ cells mainly populated the glomerular layer (GL), whereas the CR^+^ cells were mainly located in the GL and granule cell layers (GCLs). EPL, external plexiform layer; MCL, mitral cell layer; IPL, internal plexiform layer. Scale bar: 200 μm. (*H*) Quantification of CB^+^ and CR^+^ cells from both the caudal and middle regions of the OB showed no significant changes in the numbers (overall *P* = 0.41) or laminar distribution of cells (overall CR^+^− GL, *P* = 0.29; overall CR^+^− GCL, *P* = 0.98) between genotypes (overall CR^+^− GL, *P* = 0.29; overall CR^+^− GCL, *P* = 0.98). Data are shown ±SEM.

In addition, because the expression of *Visc-2* in the SVZ, RMS, and OB persists throughout postnatal development (Fig. 2), these 3 structures were examined in greater detail to determine whether neurogenesis within the adult mouse olfactory system was influenced by *Visc-2* loss. Immunohistochemistry was used to quantitatively investigate the structural integrity of the RMS, generation of neuroblasts in the SVZ, as well as neuronal fate specification and distribution within the OB—the endpoint of the newly generated neurons migrating from the SVZ. The RMS itself failed to show any change in overall positioning, size, or structure between *Visc-2*^+/+^ and *Visc-2*^−/−^ mice. The basic internal organization, with astrocyte cell processes surrounding immature migrating neuroblasts, was also unaltered as shown by the similar distribution and lack of co-localization between GFAP and Dcx immunostaining (Fig. 5*A*). Quantitative assessment of the SVZ was carried out using phosphohistone H3 (pHH3 as a marker for cell cycle progression; Ponti et al. 2013). In both genotypes, the vast majority of pHH3^+^ dividing cells were present along the lateral wall of the lateral ventricle (Fig. 5*B*). In addition, the distribution of Dcx-positive (Dcx^+^) migratory neuroblasts in this region (as well as in the corpus callosum and the striatum) was also very similar for animals of both genotypes (Fig. 5*C*). The size of the proliferative population indicated by pHH3 immunostaining was reduced in the *Visc-2*^−/−^ SVZ relative to that of the *Visc-2*^+/+^ (Fig. 5*E*), although this failed to reach statistical significance. There was also neither a significant difference in the number of pHH3^+^/Dcx^+^ double-labeled cells, nor in the proportion of the pHH3^+^ cells that were also Dcx-positive (Fig. 5*D*,*F*). To analyze neuronal fate in the OB, the distributions of 2 GABAergic interneuron populations were quantified. Neither calbindin-positive (CB^+^) cells in the glomerular layer nor calretinin-positive (CR^+^) neurons in the glomerular and granule cell layers of the OB showed a significant difference in population size or distribution between genotypes (Fig. 5*G*,*H*).

To further quantify postnatal neurogenesis, BrdU labeling was carried out using a protocol that detects newborn neurons in the OB; after 6 consecutive days of BrdU administration from P7, the OBs of *Visc-2*^−/−^ and *Visc-2*^+/+^ mice were analyzed at P28 (Supplementary Fig. 4*A*). The total number of BrdU-positive (BrdU^+^) cells in the granule cell layer was not significantly different between genotypes (Supplementary Fig. 4*A*–*C*). In addition, there was no variation in the number of BrdU^+^ cells that were also NeuN-positive by double-labeling, suggesting that there is no difference in the neuronal identity of these newly generated cells (Fig. 4*B*,*D*). Taken together, the combined OB data suggest that it is unlikely that *Visc-2* disruption adversely affects the final destination or identity of newly generated neurons from the SVZ into the OB.

**Figure 6.**
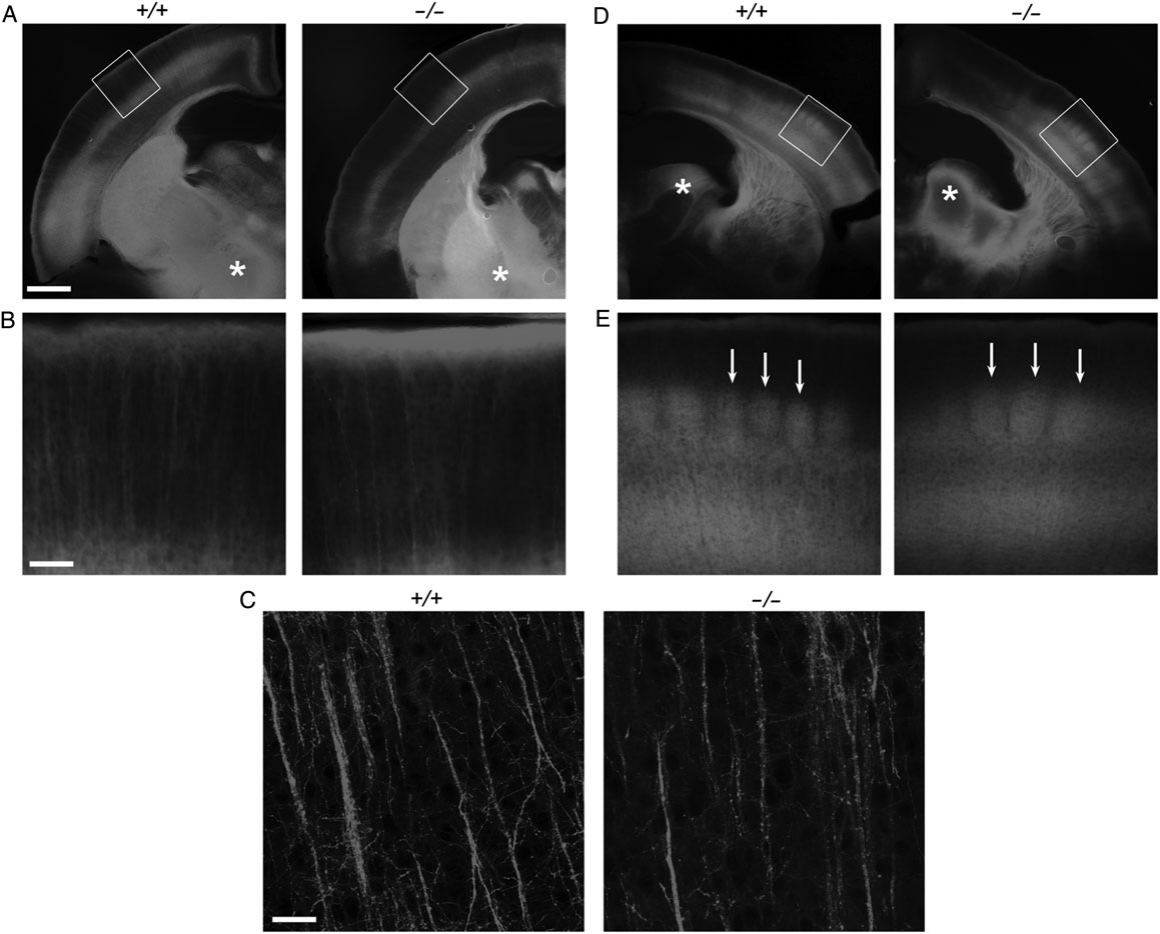
Carbocyanine dye tracing of cortical layer 5 and thalamocortical projections in *Visc-2*^−/−^ mice. P7 *Visc2*^+/+^ (+/+) and *Visc2*^−/−^ (−/−) brains were PFA-fixed and DiI crystals were placed into the cerebral peduncle (asterisks in *A*) or the ventrobasal complex of the thalamus (asterisks in *D*), followed by 6 weeks of incubation. Microscopy of coronal sections indicates that the same population of layer 5 projection neurons was backlabeled in both *Visc2*^+/+^ and *Visc2*^−/−^ brains (*A*), and both populations showed similar apical dendrites reaching to the marginal zone with a terminal tuft (*B*). The structure of the apical dendrites of the backlabeled layer 5 neurons was also similar in mice of both genotypes (*C*). Tracing from ventrobasal complex of the thalamus (asterisks in *D*) revealed thalamocortical projections reaching the cortex along similar trajectories and formed a characteristic periphery-related pattern within layer 4 of the barrel cortex in both *Visc2*^+/+^ and *Visc2*^−/−^ mice (arrows in *E*). Panels in (*B*) and (*E*) were taken from the regions indicated with boxes in (*A*) and (*D*), respectively. Scale bars: 0.5 mm in (*A* and *D*), 100 μm in (*B* and *E*), 25 μM in (*C*).

Finally, carbocyanine dye tracing experiments were carried out to examine axonal projections to the cortex at P7 from *Visc-2*^−/−^ and *Visc-2*^+/+^ mice; specifically, from the cerebral peduncle to layer 5 (Fig. 6*A*–*C*) and thalamocortical projections from the ventrobasal complex (VB) to the primary somatosensory cortex (S1) (Fig. 6*D*,*E*). These data suggest that the same population of layer 5 projection neurons with similar proportions were backlabeled in *Visc-2*^+/+^ and *Visc-2*^−/−^ brains (Fig. 6*A*). Both populations of these apical dendrites showed a comparable structural appearance and spine density as they reached the marginal zone with a terminal tuft (Fig. 6*B*,*C*). In addition, somatosensory cortex tracing demonstrated a similar pattern of thalamocortical projections in both *Visc-2*^+/+^ and *Visc-2*^−/−^ mice (Fig. 6*D*). These projections crossed the internal capsule and striatum along comparable trajectories with a similar fasciculation pattern and formed the characteristic periphery-related pattern in layer 4 of the barrel cortex (Fig. 6*E*).

**Figure 7.**
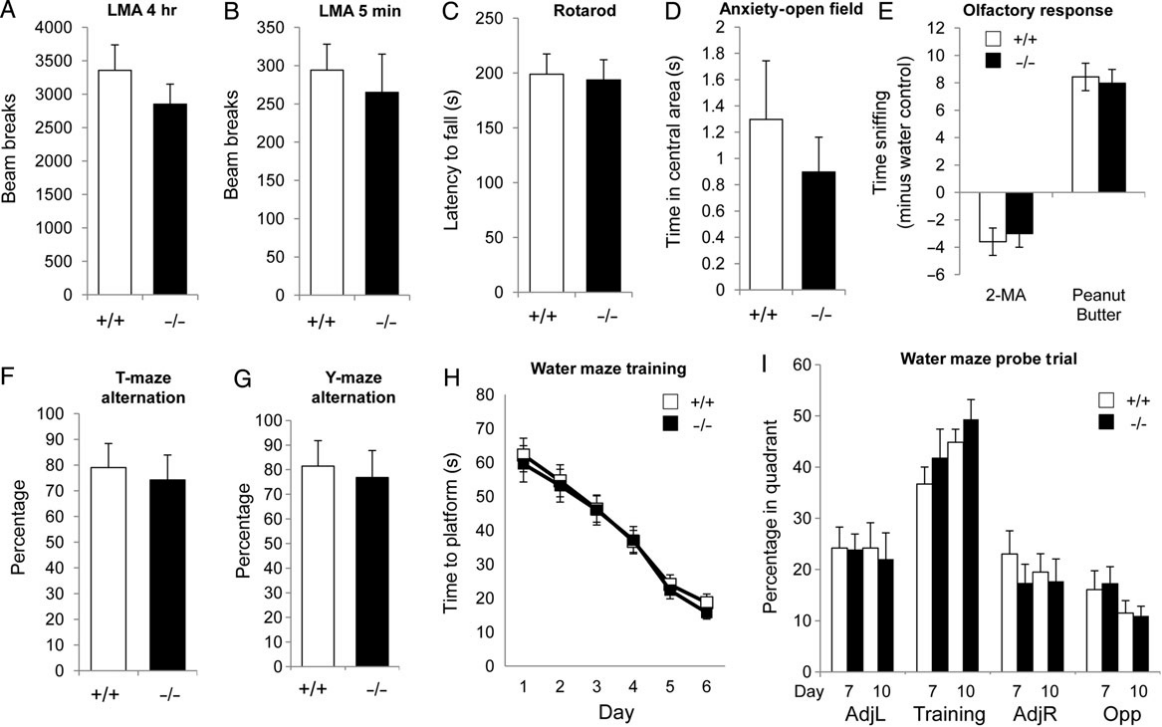
Behavioral characterization of *Visc-2* knockout mice. No significant difference in performance between adult male *Visc-2*^+/+^ and *Visc-2*^−/−^ mice (*N* = 12 for each genotype) was identified in any of the behavioral paradigms chosen. The test battery included assays of: (*A*) LMA over (*A*) 4 h (*P* = 0.57) and (*B*) 5 min (*P* = 0.31), (*C*) motor function on a rotarod (*P* = 0.85), (*D*) anxiety based on time in the center of a circular open field (*P* = 0.47), (*E*) olfactory response to aversive (2-methylbutyric acid; *P* = 0.16) or attractive (peanut butter; *P* = 0.26) scents compared with water, (*F*) short-term spatial memory in a T-maze (*P* = 0.27), and (*G*) spatial novelty in a Y-maze (*P* = 0.58). In the Morris water maze, there was no difference in (*H*), the reduction in time taken to locate the hidden platform after 6 days (genotype × day *P* = 0.49; ANOVA). (*I*) For the probe trial, the time spent in the same quadrant of the pool where the platform had been previously been located (training quadrant) compared with quadrants either side (adj L and adj R) or opposite (Opp) after 7 and 10 days of training is shown. Although mice of both genotypes spent more time in the training quadrant, there was also no significant improvement between genotypes in performance between probe trials (genotype × day *P* = 0.062; ANOVA). All data are shown ±SEM.

Although no overt pathological phenotype was evident, we continued to characterize *Visc-2*^−/−^ mice using a battery of behavioral tasks on a cohort of age- and sex-matched wild-type and knockout animals. As a baseline assessment of activity as well as exploration in a novel environment, LMA testing was performed. No significant difference in the total distance traveled throughout the trial or during the initial habituation phase was observed between *Visc-2*^+/+^ or *Visc-2*^−/−^ mice (Fig. 7*A*,*B*). There was also no difference in motor function as demonstrated by the performance on the accelerating rotarod (Fig. 7*C*), or in anxiety-related behavior as assessed in both the anxiolytic open field tests (Fig. 7*D*) and elevated plus maze (data not shown). With particular relevance to the expression of *Visc-2* in the OB, the sense of smell was assessed using an olfactory preference task where both aversive and attractive scents are presented (Witt et al. 2009). Both wild-type and knockout mice were able to clearly distinguish between the 2 classes of odors, with similar amounts of time exploring aversive versus attractive scents in both genotypes (Fig. 7*E*). We went on to analyze cognitive performance using a number of tests of spatial memory. In the short-term exploratory spatial memory tasks—spontaneous alternation and Y-maze spatial novelty preference tests—both *Visc-2*^+/+^ and *Visc-2*^−/−^ mice performed well above chance levels (Fig. 7*F*,*G*), with no significant difference in their activity in the apparatus (data not shown). In the Morris water maze test of spatial reference memory, there was no significant difference between genotypes in the rate of acquisition of the hidden platform position during 6 days of training based on swim path time (Fig. 7*H*). During the first probe trial on day 7, both *Visc-2*^+/+^ and *Visc-2*^−/−^ mice spent significantly more time in the quadrant where the platform was originally placed, which indicates that both groups of mice had learned the location of the platform to a similar extent (Fig. 7*I*). In agreement with these data, a second probe trial after 3 further training days showed a slight improvement in the time spent in the target quadrant by mice of both genotypes, with no impairment in *Visc-2*^−/−^ mice (Fig. 7*I*). Taken together, these data suggest that loss of *Visc-2* has no overt effect on brain pathology or on behavior.

## Discussion

Sequences of noncoding *Visc* transcripts are not only conserved among mammals, but expression of *Visc-2* orthologous transcripts occurs in the developing brain of marsupials, mice, and humans. Nevertheless, data from a range of neuroanatomical and behavioral analyses failed to identify physiological abnormalities in *Visc-2*^−/−^ mice compared with wild-type controls. Despite a highly localized neurodevelopmental expression pattern in the SVZ, RMS, OB, and cortex, no defects in neuronal migration or anatomy were identified in *Visc-2*^−/−^ animals, and no abnormal anxiety or cognitive behavioral phenotypes were observed.

One explanation for these data is that loss of *Visc-2* is functionally compensated for by other transcripts in the region not influenced by the *Visc-2* deletion strategy, such as the lncRNA *Visc-3*. Indeed, the transcriptional complexity of the noncoding RNA transcripts from this locus became clearer since the initiation of this study, with multiple lncRNAs as well as an mRNA all displaying specific neurodevelopmental expression profiles (Laneve et al. 2010; Shibata et al. 2011). It is noteworthy, therefore, that there was a small but significant reduction in *miR-9-2* expression in the *Visc-2*^−/−^ brain versus controls, suggesting that the deletion of the first exon of *Visc-1* has a modest diminution of transcription of the pre-mRNA9-2, which is mostly transcribed from a separate, more proximal, promoter in a neural cell line (Laneve et al. 2010). This modest reduction in *miR-9-2* expression is not expected to cause any phenotypic abnormalities, as none are reported in mice lacking this miRNA (Shibata et al. 2011). An alternative explanation for our findings is that the engineered genomic deletion of *Visc-2* has inadvertently removed a regulatory element of *miR-9-2*, such as a specific chromatin modification; indeed, there is substantial evidence for active histone methylation (e.g., H3K4me3) in the brain across the majority of the *Visc* locus (Fig. 1*C*; Stamatoyannopoulos et al. 2012). Nevertheless, the *Visc-2* deletion did not encompass the known Mef2c-binding sites upstream of the proximal pre-mRNA-9-2 promoter (Davila et al. 2014) nor did it significantly alter *Mef2c* expression levels (Fig. 4*B*).

That we were unable to identify a phenotype in *Visc-2*^−/−^ mice can be explained in 2 further ways. First, it could reflect limitations in the screening assays that were unable to detect a bona fide phenotype. We emphasize that the choice of these assays was informed by the specific expression pattern of the *Visc-2* lncRNA. Moreover, we applied a battery of behavioral, anatomical, and neuropathological tests that have been rarely, if ever, previously applied together to investigate a lncRNA knockout phenotype in the nervous system. However, it is not possible to rule out a subtle phenotype in *Visc-2*^−/−^ animals that would only become apparent at a specific time point or within a defined neuroanatomical region or after environmental manipulations, either in the laboratory or in the wild, have been applied. Neuronal hyperexcitability and seizures, for example, were only manifested in *Bc1* knockout mice upon auditory stimulation (Zhong et al. 2009). We note that there are many examples where highly evolutionarily conserved, developmentally expressed protein-coding genes have been knocked-out in the mouse, yet do not yield overt phenotypes despite considerable experimental scrutiny (Barbaric et al. 2007) and also examples of lncRNA knockout mice that fail to yield phenotypes (Nakagawa et al. 2011, 2012; Schorderet and Duboule 2011; Eissmann et al. 2012; Zhang et al. 2012; Sauvageau et al. 2013). It is also noteworthy that lncRNAs without evidence for evolutionary conservation can show phenotypes when disrupted. For example, the mouse-specific lncRNA *Braveheart* is important for cardiovascular lineage commitment (Klattenhoff et al. 2013).

Our results could also be explained if transcription of mouse *Visc-2* has no biological consequence. The *Visc-2* sequence is, however, highly conserved in human (67% identity) as well as opossum (66% based on local alignment), and its expression pattern is consistent in mouse, opossum, and human embryonic brain development which could reflect the conservation of its transcriptional regulation. Consequently, we believe it more likely that *Visc-2* is functional but that the effect of its deletion upon fitness is insufficient to manifest itself in the assays screened. One possibility is that the *Visc-2* DNA or RNA sequence acts in cis to subtly modulate the expression of nearby genes, such as *Mef2c* or *pre-miR-9-2*, or in trans to moderately alter the expression of more distal genes. Many point mutations in *Visc-2* genomic DNA appear to have been deleterious; however, as its sequence has been preferentially conserved over mammalian evolution.

Many more lncRNA knockout studies combined with phenotypic analyses at the level of detailed scrutiny applied here will be required to establish the general significance of these transcripts in the context of a whole organism. For example, it has been demonstrated that deletion of 2 large noncoding regions of approximately 1 Mb containing over a thousand conserved noncoding elements, many of which will be transcribed, does not lead to detectable abnormalities in mice (Nobrega et al. 2004), although only gross physiological assessments were performed. Therefore, it is yet to be established whether there are categories of lncRNAs that are more likely to manifest in phenotypes when disrupted in mouse. Next-generation sequencing is a potential strategy for prioritizing noncoding candidates, and a number of lncRNAs have been identified with expression patterns corresponding to specific cortical layers (Belgard et al. 2011), or in the adult mouse SVZ neural stem cell lineage (Ramos et al. 2013). It will be interesting to determine whether these transcripts play important roles in neuronal fate determination in the developing brain; consequently, a high-throughput knockout program focussing on lncRNAs, similar to that currently underway for protein-coding genes, may be required (Bradley et al. 2012). One remaining difficulty is selecting the manner by which to disrupt lncRNA transcription in a mouse model without prejudicing the outcome of whether the transcript itself, the act of transcription, or even both, transact function (Hainer et al. 2011). To date, genomic deletions to remove regions of lncRNA exons and their putative promoters have been described elsewhere in mouse (Zhang et al. 2012). Yet, as discussed above, the loss of potentially confounding chromatin modifications must be considered. Potentially, such issues can be avoided by the insertion of transcriptional termination signals (Bond et al. 2009), and recent advances in zinc-finger nuclease and CRISPR/Cas9 technology may provide a more robust high-throughput approach (Gutschner et al. 2011; Mali et al. 2013). Illustrating the importance of these methodological issues, the lncRNA *Fendrr* has been disrupted using 2 independent strategies; although both methods resulted in a lethal phenotype, *Fendrr* deletion led to delayed development of the lungs (Sauvageau et al. 2013), while multisystem defects were observed after insertion of a transcriptional termination signal (Grote et al. 2013). Whatever strategies are employed, genetic disruption of lncRNAs in whole organisms will continue to be a vital tool to dissect their functional roles and their degree of influence on development and survival.

## Supplementary Material

Supplementary material can be found at: http://www.cercor.oxfordjournals.org/.

## Funding

This work was supported by the UK Medical Research Council, Biotechnology and Biological Sciences Research Council, UK and the European Research Council through an advanced grant (DARCGENs). Funding to pay the Open Access publication charges for this article was provided by the University of Oxford.

## Supplementary Material

Supplementary Data
