# Supplementary material for: Disruption of Visc-2, a Brain-Expressed Conserved Long Noncoding RNA, Does Not Elicit an Overt Anatomical or Behavioral Phenotype
Source: Cereb Cortex. 2014 Sep 10;25(10):3572–85. doi: 10.1093/cercor/bhu196 (PMC4585502; doi:10.1093/cercor/bhu196)
Supplement: Supplementary Data [file supp_25_10_3572__index.html]

Disruption of Visc-2, a Brain-Expressed Conserved Long Noncoding RNA, Does Not Elicit an Overt Anatomical or Behavioral Phenotype — Supplementary Data 

# Disruption of *Visc-2*, a Brain-Expressed Conserved Long Noncoding RNA, Does Not Elicit an Overt Anatomical or Behavioral Phenotype

## Supplementary Data

Supplementary Data

**Files in this Data Supplement:**

- Supplementary Data - Doc file
