# Supplementary material for: Disruption of Visc-2, a Brain-Expressed Conserved Long Noncoding RNA, Does Not Elicit an Overt Anatomical or Behavioral Phenotype
Source: Cereb Cortex. 2014 Sep 10;25(10):3572–85. doi: 10.1093/cercor/bhu196 (PMC4585502; doi:10.1093/cercor/bhu196)
Supplement: Supplementary Data [file supp_bhu196_bhu196supp.doc]

**Supplementary Figure legends**

**Supplementary Figure 1**

(A) A detailed view of the multi-species sequence alignment at donor (d) and acceptor (a) splice sites of *Visc-1* and orthologous regions (canonical sites are dark green), as well as the poly-A site (dark green). (B) Conservation and relative sizes of *Visc-1* orthologues in various species. Note the conserved splice sites (green bars) in *Visc-1* exons 1, 3, 4 and 5 (alternative second exon outlined with dotted line). Grey dotted line separates zebra finch and frog putative microRNA precursor transcripts in processed and unprocessed states, respectively.
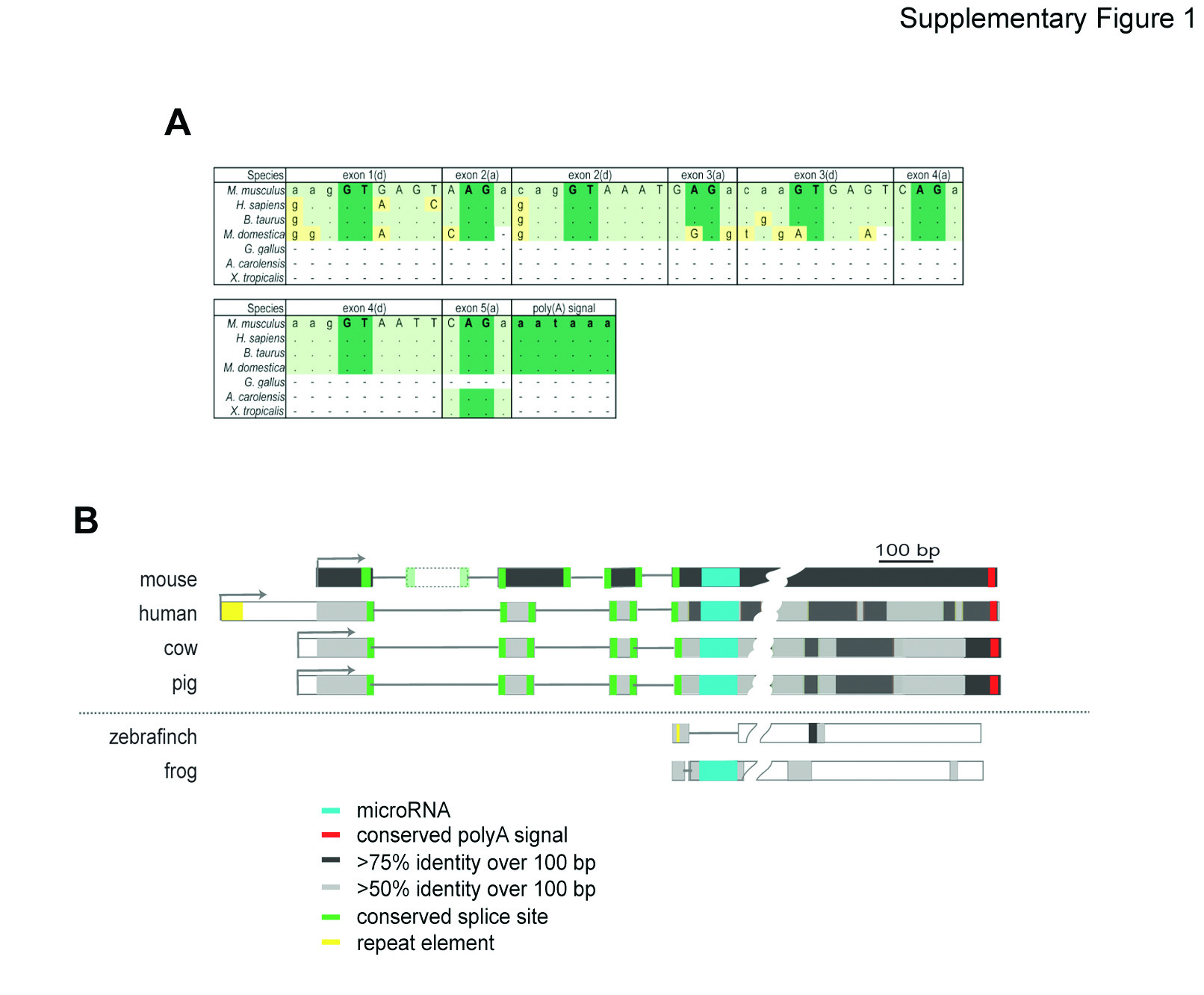


**Supplementary Figure 2**

No gross morphological abnormalities were observed in *Visc-2^-/-^* (-/-) or *Visc2^+/-^* (+/-) adult brains as compared to *Visc-2^+/+^* (+/+) as indicated by Nissl staining of equivalent coronal sections. Left to right, olfactory bulb; frontal cortex and olfactory cortex; rostral part of the parietal cortex, striatum and callosum; parietal cortex, amygdala, endopyriform cortex, internal capsule, posterior callosum and hippocampus; cerebellum. Scale bars: 2 mm
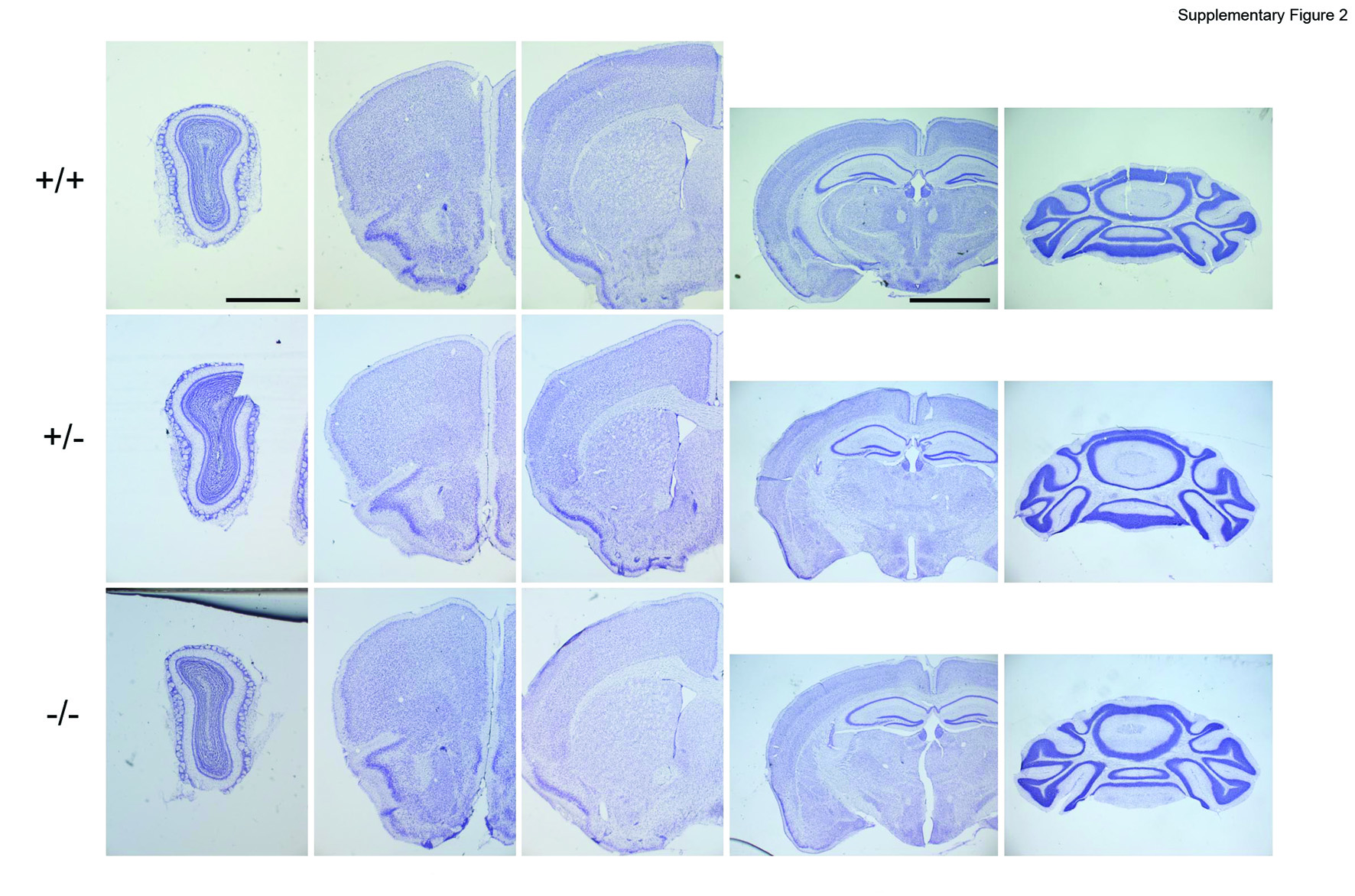


**Supplementary Figure 3**

No differences in the distribution or expression level of layer markers in the somatosensory cortex were observed between *Visc2^+/+^* (+/+) and *Visc-2^-/-^* (-/-) and mice at P1 and P56. Scale bars: 100 μM


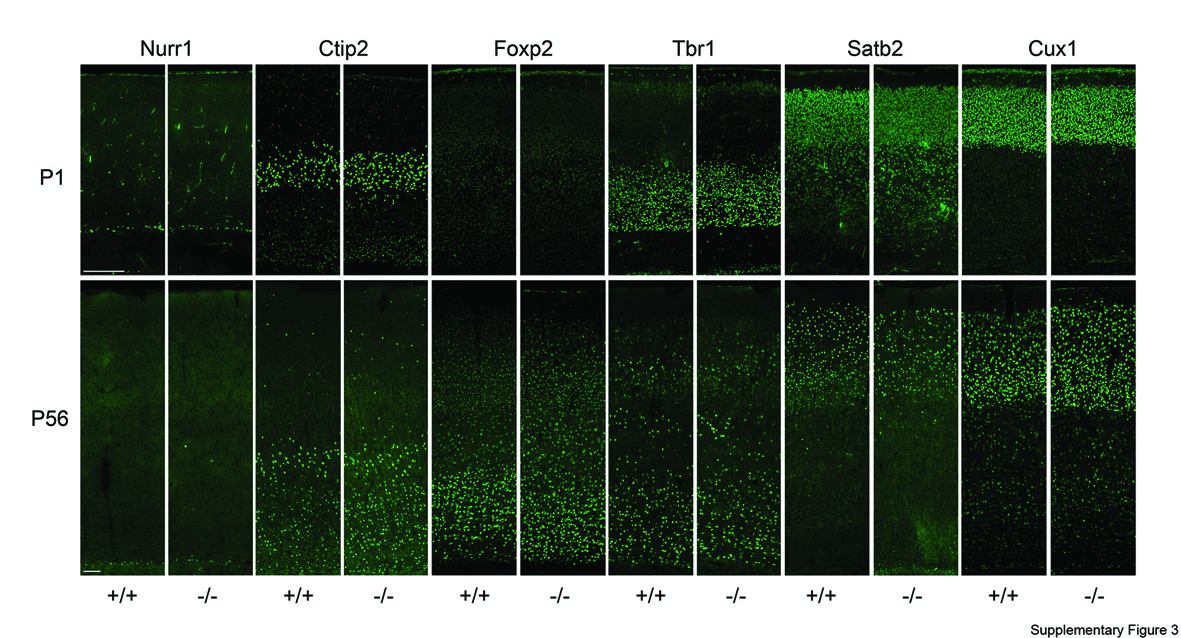


**Supplementary Figure 4**

(A) Generation of newborn cells in the OB after BrdU administration for 7 days from P7. Representative sections are shown from *Visc2^+/-^* (+/+) and *Visc-2^-/-^* (-/-) mice at P28. There was no difference in the total number of newborn BrdU positive (BrdU^+^) cells in the granule cell layer of the OB between genotypes (*P* = 0.22; (C)). There was also no difference in the number of BrdU^+^ cells that were neuronal (*P* = 0.38; (D)), as quantified using co-staining for NeuN (arrows in B) versus singly labelled BrdU^+^ cells (arrowheads in B). Data are shown +/- SEM. Scale bars: 250 μM in (A), 50 μM in (B).


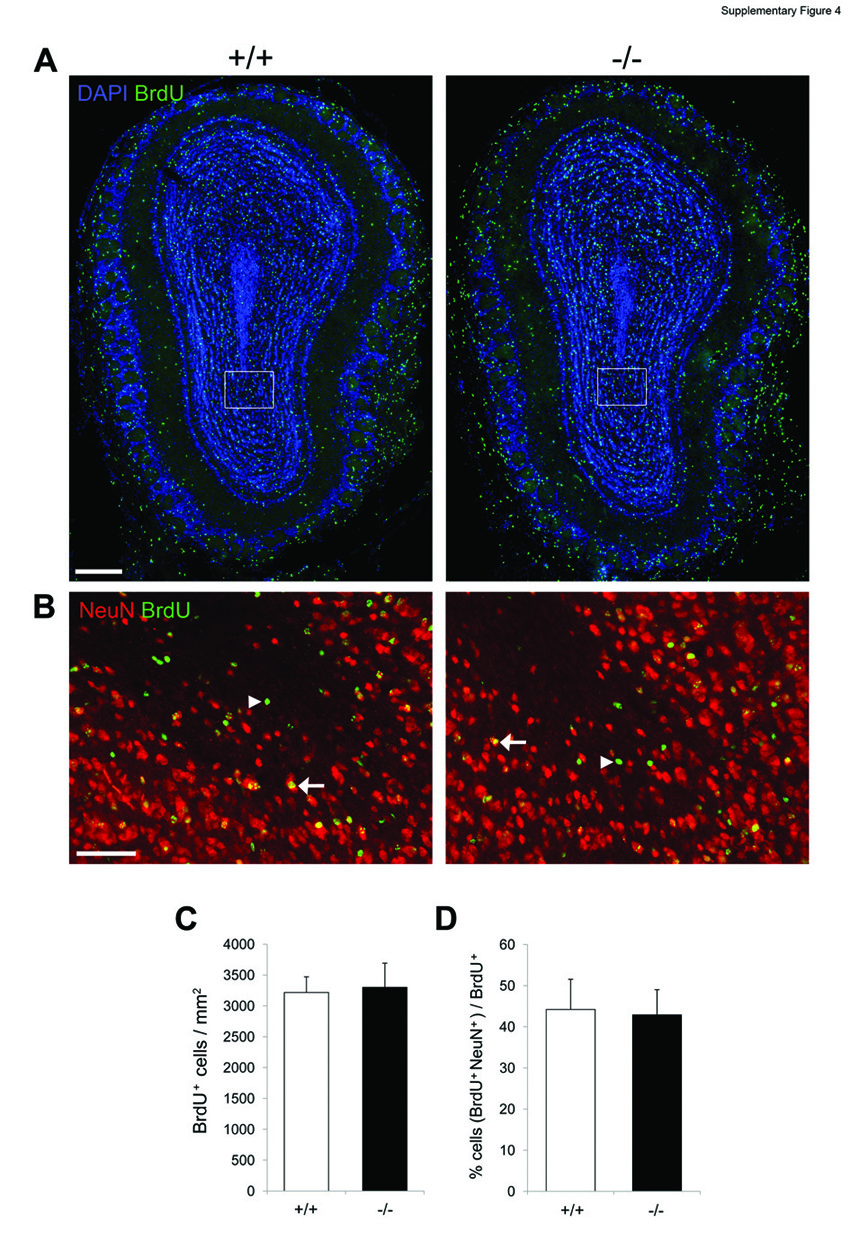


**Supplementary Table 1**

*Visc-1/-2* orthologues amongst vertebrates. Either *Visc-1* or *Visc-2* sequences were used as queries in BLASTN searches of the NCBI nr database to identify orthologous ESTs. The cut-off for significance was set at E-value < 1 x 10^-10^.

| **lncRNA** | **Species (common name)** | **Genbank accession** | **Tissue type** | **Developmental stage** |
| --- | --- | --- | --- | --- |
| *Visc-2* | *M. musculus* (mouse) | AK158494 | Visual cortex | Adult |
| *Visc-1* | *M. musculus* (mouse) | BY242621 | Visual cortex | Adult |
| *Visc-1* | *M. musculus* (mouse) | C130071C03 | Spinal cord | Adult |
|  | *R. norvegicus* (rat) | CB745429.1 | Brain | Embryo |
|  | *H. sapiens* (human) | CR599257 | Brain | Fetal |
|  | *C. lupus familiaris* (dog) | BQ290079.1 | Brain | Adult |
|  | *B. taurus* (cow) | EE241071 | Cerebellum | Fetal |
|  | *S. scrofa* (pig) | CK461258 | Pooled | Embryo |
|  | *G. gallus* (chicken) | CR405877 | Brain | Adult |

**Supplementary Table 2**

Numbers of offspring born to *Visc-2^+/-^* x *Visc-2^+/-^*pairings of each genotype.

| **Genotype** | **Male** | **Female** |
| --- | --- | --- |
| *Visc-2^+/+^* | 24 | 23 |
| *Visc-2^+/-^* | 48 | 52 |
| *Visc-2^-/-^* | 23 | 26 |
|  |  |  |
| total | 95 | 101 |
